# AI-Augmented Mentorship in Orthopedic Surgery: A Conceptual Framework for Expanding Access for Underrepresented and Less-Resourced Students

**DOI:** 10.7759/cureus.110790

**Published:** 2026-06-13

**Authors:** Joseph Salem-Hernández, Peter A Santiago-Gadea, Camilla A Pérez-Vicente, Rafael Señeriz-Ortiz, Pablo V Marrero, Norman Ramírez

**Affiliations:** 1 Department of Orthopedic Surgery, Ponce Health Sciences University, Ponce, PRI; 2 School of Medicine, Central University of the Caribbean, Bayamon, PRI

**Keywords:** artificial intelligence, diversity, health equity, large language models, medical education, mentorship, orthopaedic surgery, residency training, social media, underrepresented minorities

## Abstract

Orthopedic surgery remains one of the least diverse medical specialties in the United States, with women and racial/ethnic minorities being significantly underrepresented. Traditional mentorship models often depend on institutional prestige, geographic proximity, and professional networks, creating barriers for underrepresented minority students and trainees from lower-resourced institutions. Increasing residency competitiveness further highlights disparities in mentorship access.

The aim of this study was to examine how artificial intelligence (AI) technologies may expand mentorship access and reduce disparities in orthopedic surgery training pathways. This conceptual framework paper narratively synthesizes published literature on mentorship disparities in surgical training, AI applications in medical education, and residency selection equity, identified through database searches (PubMed, ERIC, Google Scholar) using terms including "orthopaedic surgery", "mentorship", "artificial intelligence", "large language models", "residency diversity", and "underrepresented minorities", supplemented by hand-searching reference lists of key articles. Three AI-driven approaches are integrated: (1) large language model (LLM)-based virtual mentorship platforms, (2) residency data dashboards, and (3) social media analytics for network mapping.

AI-augmented mentorship platforms may improve accessibility, scalability, and transparency compared with traditional mentorship models. LLM-based tools provide continuous access to educational support, while dashboards and social media analytics may reduce informational and networking barriers. However, these are proposed benefits extrapolated from adjacent fields; none have been empirically validated in an orthopedic surgery context. Risks including algorithmic bias, unequal technology access, and limited psychosocial support must be addressed. Equity-centered implementation, diverse training datasets, human oversight, and bias monitoring are essential prerequisites to ensure these technologies reduce rather than worsen disparities.

AI technologies have the potential to broaden mentorship access in orthopedic surgery, though this remains unproven empirically and requires prospective validation before programmatic adoption. Strategic integration of AI-based mentorship tools may help complement, rather than replace, traditional human mentorship. Rigorous evaluation, equity-centered design, human oversight, and strong data governance are essential. Collaboration among orthopedic organizations, medical schools, and residency programs will be necessary to ensure equitable implementation.

## Introduction and background

Orthopedic surgery stands at a critical juncture in addressing workforce diversity. Despite decades of pipeline initiatives, the specialty remains one of the least diverse in American medicine. Women constitute only 15.3% of orthopedic surgery residents and fellows, while racial and ethnic minorities remain significantly underrepresented relative to the general population [1]. These disparities are not merely statistical artifacts but reflect systemic inequities with profound implications for patient care, innovation, and the profession's future.

Before proceeding, key terms warrant definition. Mentorship refers to an ongoing developmental relationship involving career guidance, psychosocial support, and direct advocacy. Sponsorship refers to active public advocacy on behalf of a mentee, including letters of recommendation and direct introductions to program directors and colleagues, and it is the most critical and least replicable function of human mentorship. URM (underrepresented in medicine) refers to racial and ethnic groups underrepresented in the medical profession relative to their proportions in the U.S. general population, as defined by the Association of American Medical Colleges. Social capital refers to the professional networks, relationships, and institutional affiliations that facilitate access to career opportunities. Lower-resourced institutions refer to medical schools and training programs with limited orthopedic faculty, no home residency program, limited research infrastructure, or limited alumni networks in the specialty, which is a description of structural resource constraints rather than a ranking judgment.

The competitive landscape of orthopedic surgery residency selection intensifies these challenges. In 2024, approximately 40% of all applicants, including U.S. MD seniors, DO graduates, and international medical graduates, to orthopedic surgery residency programs went unmatched, reflecting the most competitive match rates among all surgical specialties [2]. Unmatched rates are substantially higher among DO applicants and international medical graduates than among U.S. MD seniors. This intense competition disproportionately affects students from underrepresented backgrounds and those at lower-resourced institutions, who often lack access to the mentorship networks, research opportunities, and insider knowledge that facilitate successful residency applications [3]. Traditional mentorship models, which rely heavily on geographic proximity, institutional prestige, and pre-existing social networks, inadvertently perpetuate these disparities by favoring students with access to elite academic medical centers and established professional connections [4].

Mentorship serves multiple critical functions in medical training, including career guidance, research collaboration facilitation, psychosocial support, and sponsorship through direct advocacy and professional network access [5]. It is important to distinguish among three related but distinct barriers: mentorship deficits (limited access to guidance, career counseling, and research support), sponsorship deficits (limited advocacy through letters of recommendation, introductions, and direct endorsements), and hostile or noninclusive learning environments (implicit bias, microaggressions, and unwelcoming training climates). These co-occur but require different interventions, and artificial intelligence (AI) tools address only the first. The absence of effective mentorship creates cascading disadvantages. Students without mentors are less likely to engage in orthopedic research, attend national conferences, secure away rotations at competitive programs, or receive strong letters of recommendation, all of which significantly influence residency match success [6]. For URM students and those at lower-resourced institutions, these barriers are compounded by implicit bias in application evaluation, limited representation of same-identity mentors, and financial constraints that restrict participation in traditional networking opportunities [7].

AI technologies offer opportunities to address informational and geographic barriers in mentorship access. LLMs such as GPT-4, Claude, and Google's Med-PaLM 2 can provide 24/7 access to career guidance, research support, and clinical knowledge [8]. The American Academy of Orthopaedic Surgeons (AAOS) is the specialty's primary professional society; the Accreditation Council for Graduate Medical Education (ACGME) accredits U.S. residency programs; and the National Resident Matching Program (NRMP) administers the algorithm-based match process that assigns applicants to residency positions. Data dashboards can enhance transparency regarding residency program diversity metrics, match outcomes, and training opportunities, leveling the informational playing field for all applicants [9]. Social media analytics can identify mentorship opportunities and professional networks that transcend institutional boundaries [10]. These technologies do not replace human mentorship but rather augment traditional models by addressing specific barriers, including geographic isolation, information asymmetry, and limited network access, that disproportionately affect underrepresented students. Critically, AI cannot address structural inequity in residency selection that involves evaluation bias, financial barriers, prestige effects, and underrepresentation of same-identity mentors, all of which require organizational and cultural interventions beyond what technology can provide.

This conceptual framework paper examines how AI-augmented mentorship may expand access for URM students and those at lower-resourced institutions pursuing orthopedic surgery. We present a framework integrating LLM-based virtual mentorship, residency transparency dashboards, and social media network mapping. We analyze the plausible benefits, limitations, and ethical considerations of these approaches, with particular attention to ensuring that technological solutions advance rather than undermine equity goals. This is a conceptual framework proposal, not a report of empirical findings from an implemented program. Our purpose is to provide orthopedic educators, program directors, and national organizations with evidence-informed strategies for developing and evaluating AI tools to support a more diverse and inclusive workforce.

Disparities in orthopedic surgery mentorship

The underrepresentation of women and racial/ethnic minorities in orthopedic surgery reflects systemic barriers that begin early in medical training and persist throughout career trajectories. Women comprise only 15.3% of orthopedic surgery residents and fellows, the lowest percentage among all surgical specialties [1]. Racial and ethnic minorities face similar disparities, with Black, Hispanic, and Native American physicians significantly underrepresented relative to their proportion in the general population [11]. Systematic reviews of diversity interventions in surgical training confirm that mentorship access disparities significantly disadvantage these groups [11]. These disparities have remained stubbornly persistent despite decades of pipeline programs and diversity initiatives.

The consequences of mentorship disparities are measurable and significant. Students without mentors are less likely to engage in orthopedic research, with one study documenting that research productivity, measured by publications, presentations, and grants, strongly correlates with mentorship access and institutional resources [12]. Research productivity, in turn, significantly influences residency match success in orthopedic surgery, creating a self-perpetuating cycle where students with limited mentorship access face compounded disadvantages in the competitive residency application process. Geographic isolation further exacerbates these disparities. Students at institutions without orthopedic surgery residency programs or those in rural areas have limited opportunities for direct interaction with orthopedic faculty, reducing exposure to the specialty and limiting access to the informal networks that facilitate career advancement [12].

Gender-specific barriers compound these challenges for women pursuing orthopedic surgery. A survey-based analysis of male Indian orthopedic surgeons revealed persistent attitudes questioning female colleagues' competence and suitability for the specialty, highlighting how implicit bias creates hostile training environments that deter women from entering and remaining in the field [13]. While this study examined attitudes outside the United States, similar patterns of implicit bias and hostile environments have been documented in U.S. surgical training contexts [7,11]. Similarly, analysis of leadership positions in spinal societies worldwide documented significant gender inequity, with women holding disproportionately few leadership roles despite increasing representation in training programs [14]. These findings suggest that mentorship alone is insufficient without concurrent efforts to address systemic bias and create inclusive training environments, which is a structural challenge that AI tools cannot resolve.

The COVID-19 pandemic further disrupted traditional mentorship models, eliminating in-person away rotations and conference networking opportunities that historically served as critical mechanisms for students to demonstrate interest and build relationships with potential mentors [15]. While virtual alternatives emerged, they disproportionately disadvantaged students with limited digital literacy, unreliable internet access, or competing caregiving responsibilities, factors that disproportionately affect URM students and those from lower socioeconomic backgrounds. It is important to note, however, that some well-designed virtual programs successfully expanded access for students who would otherwise have been excluded by geographic or financial constraints [16]. The pandemic thus highlighted both the fragility of traditional mentorship models and the conditional potential of technology-enabled alternatives to expand access when thoughtfully designed.

Social capital and the hidden curriculum

Social capital - the networks, relationships, and institutional affiliations that facilitate access to opportunities and resources - plays a decisive role in orthopedic surgery career advancement. Students at elite medical schools benefit from established relationships between their institutions and prestigious residency programs, faculty connections to national leaders in the field, and peer networks that provide insider knowledge about application strategies and program cultures. This social capital operates largely through informal channels, constituting what medical education scholars term the "hidden curriculum," the unwritten rules, expectations, and knowledge that are transmitted through observation and informal mentorship rather than formal instruction.

The hidden curriculum in orthopedic surgery encompasses several critical domains. First, it includes knowledge about which research topics, methodologies, and publication venues are valued by residency selection committees. Students with mentors embedded in the orthopedic community receive guidance on selecting research projects that align with current priorities, identifying collaborators who can facilitate high-impact publications, and navigating authorship conventions. Second, the hidden curriculum includes tacit knowledge about residency program selection: which programs are receptive to applicants from specific backgrounds, how to interpret program websites and interviews to assess culture and training quality, and strategies for demonstrating genuine interest without appearing overeager. Third, it encompasses professional socialization, learning the communication styles, behavioral norms, and professional identities valued within orthopedic surgery.

Students lacking access to this hidden curriculum face significant disadvantages. Without mentors to decode implicit expectations, they may invest time in research projects that do not enhance their competitiveness, apply to programs where they are unlikely to match, or inadvertently violate unwritten norms during interviews and interactions with faculty. These disadvantages are particularly acute for first-generation medical students who cannot rely on family members' professional networks or experiential knowledge to navigate the application process. Similarly, URM students may lack access to same-identity mentors who can provide guidance on navigating bias and microaggressions in training environments where they are underrepresented.

LLM-based tools can decode and explain elements of the hidden curriculum, providing guidance on research topic selection, authorship norms, program culture interpretation, and interview conduct, thereby reducing informational disadvantages that accrue to students without well-connected mentors. However, AI cannot replicate the trust-based elements of the hidden curriculum that operate through personal relationships: it cannot write a credible letter of recommendation, introduce a student to a program director, or sponsor a candidacy through direct professional advocacy. The distinction between informational access and relational sponsorship is fundamental to understanding what AI can and cannot achieve.

Evidence suggests that residency programs preferentially select applicants from top-ranked medical schools, both because these students often have stronger objective credentials and because program directors have established relationships with faculty at these institutions who can provide detailed, credible assessments of applicants [3]. It is important to note, however, that institutional prestige is one of several interacting factors, alongside board examination scores, research productivity, letters of recommendation, clinical performance, and away rotation performance, that influence match outcomes. Presenting institutional prestige as the sole driver would overstate its role and obscure other available interventions.

Networking opportunities further concentrate social capital among already-advantaged students. National orthopedic conferences, visiting professorships, and away rotations provide critical opportunities to build relationships with potential mentors and demonstrate interest to residency programs. However, these opportunities require significant financial investment, including conference registration fees, travel costs, and accommodation expenses, which create barriers for students from lower socioeconomic backgrounds [5]. Even when financial support is available, students with caregiving responsibilities or those who must work to support themselves during medical school may be unable to participate in these networking opportunities, further limiting their access to social capital.

The digital divide and AI opportunity

The digital divide, defined as disparities in access to technology, internet connectivity, and digital literacy, represents both a challenge and an opportunity in efforts to democratize mentorship access. While AI technologies offer potential to provide scalable, accessible mentorship resources, their benefits will accrue primarily to students who have reliable internet access, appropriate devices, and the digital literacy to effectively utilize these tools [16]. Without intentional efforts to address the digital divide, AI-augmented mentorship may exacerbate existing inequities by providing additional advantages to already-privileged students while leaving behind those with limited technological access.

Data on the digital divide in medical education reveal significant disparities. A study of medical students during the COVID-19 pandemic found that students from rural areas, lower socioeconomic backgrounds, and underrepresented minority groups were significantly more likely to report inadequate internet connectivity, lack of appropriate devices for virtual learning, and competing demands such as caregiving responsibilities that limited their ability to engage with online educational resources [16]. These disparities translated into measurable differences in academic performance and professional development opportunities.

Digital literacy - the skills required to effectively navigate, evaluate, and utilize digital technologies - represents an additional dimension of the digital divide. A critical misconception is that younger students are inherently "digital natives" who can effectively use AI tools without training. Research demonstrates significant variation in digital literacy that correlates with socioeconomic background, educational experiences, and prior exposure to technology [17]. Effective use of AI tools such as LLMs requires specific skills: crafting effective prompts, critically evaluating AI-generated outputs for accuracy and bias, recognizing hallucinations, and integrating AI assistance with human judgment. These are learned competencies, not generational endowments. Students without prior exposure to these technologies or without mentors who can model effective use may receive worse or misleading guidance even when they have device and connectivity access.

Addressing the digital divide requires concrete institutional action, specifically: (1) subsidized device and broadband access programs at medical schools serving higher proportions of URM students; (2) mobile-compatible, offline-capable AI tool interfaces; (3) multilingual materials and accessibility features; (4) structured AI literacy curricula covering prompt design, output verification, bias recognition, and appropriate escalation to human mentors; and (5) human technical support staff, particularly at lower-resourced institutions. Critically, AI mentorship tools should be institutionally provided rather than student-purchased. Requiring students to independently identify, access, and pay for AI tools reproduces the resource inequity these tools are intended to address. National organizations and medical schools should provide standardized, vetted AI mentorship tools as part of institutional support infrastructure.

The COVID-19 pandemic accelerated the adoption of digital technologies in medical education, demonstrating both the feasibility and limitations of technology-enabled learning and mentorship. Virtual summer research programs, such as the remote program for URM students pursuing orthopedic surgery described by Hastings et al., successfully provided structured mentorship and research exposure to students who would have been unable to participate in traditional in-person programs due to geographic or financial constraints [16]. Participants reported high satisfaction and subsequently matched into orthopedic surgery residencies at rates comparable to students who participated in traditional in-person programs, suggesting that virtual mentorship can effectively support career development when thoughtfully designed and implemented.

Emerging evidence on AI applications in medical education further supports the potential for technology-augmented mentorship. A study of generative AI for electrocardiogram interpretation education in nursing students found that AI-enabled personalized, adaptive learning improved student performance and confidence compared to traditional didactic instruction [8]. Similarly, research on LLM performance in clinical decision support has demonstrated that these models can provide accurate, evidence-based guidance on complex medical questions, though with important limitations regarding hallucination risk and the need for human oversight. These findings suggest that AI tools, when appropriately designed and implemented with human oversight, can effectively augment traditional educational and mentorship models [18-26].

## Review

Conceptual framework and methods

This conceptual framework paper narratively synthesizes evidence from medical education literature, emerging research on AI applications in healthcare training, and analysis of mentorship disparities in surgical specialties. Searches were performed in PubMed, ERIC, and Google Scholar using the following terms: "orthopaedic surgery diversity", "mentorship disparities", "residency selection equity", "artificial intelligence medical education", "large language models clinical", "social capital hidden curriculum", and "digital divide medical students". Searches were conducted between January and April 2026 and were limited to English-language publications. We included peer-reviewed studies, review articles, program evaluations, and policy analyses; we excluded conference abstracts without full publication. Reference lists of key articles were hand-searched. Consistent with narrative review methodology, articles were selected based on relevance to the three framework components rather than through formal screening protocols. Because this is a conceptual framework paper rather than a systematic review, PRISMA-style reporting is not applicable; however, we have aimed to be transparent about our search approach so that readers can assess the evidentiary basis for each recommendation.

The framework integrates three complementary approaches: LLM-based virtual mentorship, data dashboards for residency program transparency, and social media analytics for network mapping. Each approach addresses specific barriers to mentorship access while acknowledging inherent limitations that require human oversight and complementary traditional mentorship.

LLM-based virtual mentorship

LLMs represent a transformative technology for expanding mentorship access by providing 24/7 availability, scalability to serve thousands of users simultaneously, and personalized responses to individual queries. LLMs such as GPT-4, Claude, and Google's Med-PaLM 2 have demonstrated capability to provide accurate and contextually appropriate guidance on a wide range of medical education topics, ranging from career planning and research methodologies to clinical knowledge and application strategies [18]. Table 1 summarizes the primary LLM platforms, their functions, evidence base, status, and limitations for medical education and orthopedic mentorship applications.

**Table 1:** Summary of AI tool categories, implementations, and evidence base in medical education and orthopedic mentorship Evidence level definitions: Emerging = case series, pilot studies, or early-phase trials only; Observational = cross-sectional or cohort studies without randomization; Pilot RCT = one or more randomized pilot trials without definitive trials; Commercially available = active platform without diversity-specific validation; Conceptual/proposed = no published implementation data. Status definitions: Existing = commercially available or published implementation; Proposed = recommended by authors, not yet implemented; Adapted = evidence from adjacent medical fields AAOS, American Academy of Orthopaedic Surgeons; AI, artificial intelligence; AMA, American Medical Association; LLM, large language model; RCT, randomized controlled trial; URM, underrepresented minority

AI Tool Category	Platform / Implementation	Primary Function	Target User	Evidence Level	Status	Key Limitation
LLM	ChatGPT-4 / GPT-4o	24/7 virtual mentorship, Q&A, career guidance	Medical students, residents	Emerging (case series, pilot studies)	Existing	Hallucination risk; lacks clinical nuance
LLM	Google Gemini / Med-PaLM 2	Clinical knowledge support, exam preparation	Pre-residency applicants	Emerging	Existing	Not validated for orthopedic-specific content
LLM	Claude (Anthropic)	Long-context document review, research support	Applicants, researchers	Emerging	Existing	Requires prompt engineering expertise
LLM-integrated chatbot	Multiprofessional AI-Integrated Clinical Chatbot [19]	Risk estimation, triage, clinical decision support	Trainees, educators	Pilot RCT evidence	Adapted from adjacent fields	Requires institutional oversight
Data dashboard	AAOS Residency Transparency Portal	Program diversity metrics, match outcomes, faculty demographics	Applicants, program directors	Conceptual / pilot	Proposed (author-recommended; does not currently exist)	Data standardization challenges
Data dashboard	Doximity / Residency Explorer [9]	Residency program comparison, board scores, fellowship placement	Medical students	Commercially available	Existing	Limited diversity-specific metrics
Data dashboard	FREIDA Online (AMA) [9]	Program-level training data, duty hours, salary	Applicants	Commercially available	Existing	Infrequently updated; self-reported data
Social media analytics	Twitter/X network analysis [9,20]	Mentor identification, hashtag community mapping (#Orthotwitter)	Students, residents, faculty	Observational studies	Existing	Platform algorithm bias; not representative of all surgeons
Social media analytics	LinkedIn professional network mapping [20]	Cross-institutional mentor connections, career trajectory analysis	Applicants, faculty	Observational	Existing	Underrepresentation of URM surgeons; algorithm-driven filtering
Social media analytics	Instagram / TikTok content analysis [9]	Residency program culture, wellness representation	Medical students	Descriptive studies	Existing	Curated content may misrepresent culture
Remote mentorship platform	Virtual summer research programs (Hastings et al.) [16]	Structured mentorship for URM students, research exposure	URM pre-med / medical students	Pilot program	Existing (pilot scale)	Scalability; requires faculty time investment
AI-powered matching	Algorithm-based mentor-mentee matching	Compatibility scoring based on research interests, demographics	Applicants	Theoretical / pilot	Proposed	Algorithmic bias; limited validation

Career Guidance and Application Strategy

LLMs can address specific informational disadvantages associated with the lack of mentorship access. Examples of how a student might use an LLM-based mentorship tool include drafting an outreach email to a potential research mentor, identifying orthopedic surgery residency programs with strong diversity, equity, and inclusion (DEI) records, outlining a plan for securing an away rotation, reviewing a personal statement for clarity and tone, developing a research question based on a clinical observation, comparing program characteristics using standardized dashboard data, and identifying board examination preparation resources. For each of these tasks, the LLM provides a starting point, whether a draft, a list, or a framework, that the student then refines with human guidance.

LLM performance in clinical decision support has demonstrated substantial concordance with expert recommendations, though hallucination risk and the need for human oversight remain important limitations [19]. Students should be explicitly taught that LLMs are prone to hallucination (generating plausible but incorrect information), may reflect training data that is outdated or biased, and can produce recommendations that reflect historical underrepresentation of URM surgeons in published mentorship literature. High AI-expressed confidence does not indicate accuracy. All LLM-generated career guidance requires human verification before acting on it.

A particular risk in AI-augmented mentorship is decision offloading, the tendency for students to accept AI-generated recommendations such as program rank lists, outreach targets, and research directions as authoritative rather than as inputs for reflection and human dialogue. Students should be explicitly instructed that AI-generated career guidance is a population-level approximation, not personalized mentorship, and that any high-stakes decision, including program list construction, letter of recommendation requests, and research commitments, must be reviewed with a human mentor before action.

Implementation Considerations and Limitations

Successful implementation of LLM-based virtual mentorship requires addressing several critical limitations. First, hallucination risk necessitates human oversight and user education on critically evaluating AI outputs. Students must be taught to verify factual claims, cross-reference recommendations with authoritative sources, and recognize the limitations of AI-generated guidance. Second, LLMs lack the relational depth and contextual understanding that characterize effective human mentorship. They cannot provide the psychosocial support, emotional validation, or personalized encouragement that are essential components of mentorship relationships [21]. Third, LLMs cannot provide sponsorship - the direct advocacy and professional network access that human mentors offer through letters of recommendation, introductions to colleagues, and explicit endorsements of mentees' capabilities. These limitations underscore that LLM-based mentorship must augment rather than replace human relationships; Table 2 maps both AI capabilities and required human complements across key dimensions [5,22].

**Table 2:** Barriers to mentorship access for underrepresented minority students and students at lower-resourced institutions, and corresponding AI-based solutions Note: "Potential Impact" ratings reflect plausible impact based on evidence from adjacent fields; none have been empirically validated in orthopedic surgery contexts. AI, artificial intelligence; DEI, diversity, equity, and inclusion; LGBTQ+, lesbian, gay, bisexual, transgender, queer/questioning, and other identities; LLM, large language model; URM, underrepresented minority

Barrier Domain	Specific Barrier	Affected Population	Proposed AI Solution	AI Tool	Potential Impact	Required Human Complement	Ref
Geographic	Limited access to academic medical centers with orthopedic faculty	Students at community / regional schools	Virtual LLM-based mentorship; remote video platforms	LLM, teleconferencing	High impact: may substantially reduce geographic barriers, though internet reliability remains a prerequisite	Human mentor relationships; away rotation facilitation; letters of recommendation	[16]
Institutional prestige	Fewer research opportunities; lower faculty-to-student ratios	Lower-resourced institutions	AI-assisted research support; automated literature review	LLM (ChatGPT, Claude)	High impact: may democratize access to research guidance, though faculty collaboration remains necessary	Faculty mentorship for research design, authorship, and publication strategy	[18]
Social capital	Lack of established networks; fewer insider connections	URM students, first-generation students	Social media analytics; network mapping tools	Twitter/X, LinkedIn analytics	Moderate impact: identifies potential mentors but does not guarantee meaningful mentorship or sponsorship	Human follow-through, relationship-building, and sponsorship cannot be replaced by AI	[20]
Financial	Cost of visiting rotations, conferences, interview travel	URM, lower socioeconomic status students	Virtual interview prep; AI-simulated mock interviews	LLM-based interview simulation	Moderate impact: reduces but does not eliminate cost burden	Financial aid advocacy; institutional funding for away rotations	[5]
Information asymmetry	Opaque residency program selection criteria and culture	All applicants; disproportionately affects URM students	Data dashboards with standardized program metrics	Residency transparency dashboards	High impact: levels informational playing field, though data completeness requires ongoing verification	Human interpretation of program fit; direct resident contact for cultural assessment	[22]
Implicit bias	Biased evaluation of applications from underrepresented groups	URM students	Blind AI screening tools; structured evaluation rubrics	AI-assisted application review	Moderate impact: risk of encoding existing bias; requires continuous auditing	Human DEI training; structured holistic review processes	[23]
Time zone / schedule	Difficulty scheduling mentorship across time zones	International, rural students	Asynchronous LLM-based Q&A; recorded guidance modules	LLM platforms	High impact: enables asynchronous mentorship, though synchronous human interaction remains valuable	Regular scheduled check-ins with human mentors	[16]
Representation deficit	Lack of same-identity mentors in orthopedic surgery	URM, women, LGBTQ+ students	Social media analytics to identify diverse mentors	LinkedIn, Twitter analytics	Moderate impact: identification does not ensure engagement	Specialty society programs to increase diverse faculty engagement	[10]
Digital literacy	Variable comfort with AI tools and digital platforms	First-generation, lower socioeconomic background students	Structured onboarding; tiered AI tool tutorials; AI literacy curricula	Educational platforms	Moderate impact: requires dedicated training; "digital native" status does not guarantee AI literacy	Human AI literacy instructors; peer mentorship in AI tool use	[17]

Data dashboards for residency program transparency

Information asymmetry, meaning unequal access to knowledge about residency programs, selection criteria, and match outcomes, represents a significant barrier to equitable residency selection. Students at elite medical schools benefit from insider knowledge transmitted through faculty mentors, alumni networks, and peer experiences, while students at lower-resourced institutions must rely on limited publicly available information that often fails to capture critical aspects of program culture, training quality, and receptivity to applicants from diverse backgrounds. Data dashboards that aggregate and standardize residency program information can level this informational playing field, providing all applicants with transparent access to metrics that inform program selection decisions. It is important to note that not all residency transparency tools require AI; data aggregation dashboards are primarily digital transparency tools, and AI is additive, enabling natural-language query interfaces and automated anomaly detection, but is not required for a functional transparency platform.

Diversity and Inclusion Dashboard Metrics

Proposed diversity metrics include resident and faculty URM and gender percentages, historical match rates by applicant background, and program-specific DEI initiatives. Training quality metrics should cover case volumes, board examination pass rates, fellowship placement, research productivity, and resident wellness indicators. Together, these data reduce reliance on institutional prestige as a proxy for program quality, with transparency itself creating incentives for programs to improve [22]. A comprehensive transparency dashboard would integrate data from multiple existing sources: ACGME program outcome reports, NRMP match data stratified by applicant demographics and program (where publicly available), FREIDA and Doximity Residency Explorer, and applicant experience surveys administered through the Electronic Residency Application Service (ERAS). Data accuracy would require mandatory standardized reporting rather than voluntary self-report, third-party auditing, and confidence intervals for small-cell data.

Implementation Challenges and Solutions

Implementing residency transparency dashboards faces several significant challenges. First, data standardization requires agreement among programs on definitions, measurement methods, and reporting timelines. The ACGME and specialty boards such as the American Board of Orthopedic Surgery (ABOS) would need to establish standardized reporting requirements and provide technical infrastructure for data collection and dissemination. Second, programs may resist transparency, particularly regarding diversity metrics and match outcomes, due to concerns about competitive disadvantage or reputational risk. Addressing this resistance requires leadership from national organizations such as AAOS and the American Orthopaedic Association (AOA) to establish transparency as a professional norm and ethical obligation. Third, programs with few URM residents in a given year may be identifiable from disaggregated match outcome data; aggregate reporting with suppression of cells with fewer than five individuals is necessary to protect privacy. Fourth, transparency dashboards create incentives for programs to not only improve reported metrics but also create incentives for performative reporting, which is improving numbers without changing practice. Mitigation requires third-party data verification, longitudinal tracking to detect pattern reversals, and qualitative data such as resident experience surveys that capture culture rather than only demographic counts. Table 3 outlines a recommended implementation framework.

**Table 3:** Recommended implementation framework for AI-augmented mentorship programs in orthopedic surgery *Timeline caveat: these are aspirational targets contingent on organizational commitment, data-sharing agreements, FERPA/HIPAA compliance resolution, and sustained funding. Phase 2 piloting must include a formal equity impact assessment and usability evaluation before national scaling is recommended in Phases 3 and 4. AAOS, American Academy of Orthopaedic Surgeons; ACGME, Accreditation Council for Graduate Medical Education; AHRQ, Agency for Healthcare Research and Quality; AI, artificial intelligence; AMA, American Medical Association; FERPA, Family Educational Rights and Privacy Act; GME, graduate medical education; HIPAA, Health Insurance Portability and Accountability Act; LLM, large language model; NIH, National Institutes of Health; NOMA, National Osteopathic Medical Association; SNMA, Student National Medical Association; URM, underrepresented minority

Phase	Recommendation	Responsible Stakeholder	Timeline*	Measurable Outcome	Funding Source	Priority
Phase 1: Foundation	Establish standardized residency program diversity data reporting (URM resident %, faculty diversity, match outcomes by race/ethnicity)	AAOS, ACGME, Program Directors	0-12 months	≥80% program participation in data reporting; validated data submission protocol published	AAOS dues; federal DEI grants	High
	Develop equity-centered AI tool design guidelines for medical education applications	AAOS Diversity Committee, AI developers	0-12 months	Published consensus guidelines; ≥3 national organizations endorsing standards	Specialty society funds; philanthropy	High
	Create URM student digital literacy curriculum for AI tool utilization	Medical schools, SNMA, NOMA	0-12 months	Pre/post digital literacy assessment scores; ≥15-point improvement on validated scale	Medical school GME offices; federal grants	High
Phase 2: Pilot	Launch LLM-based virtual mentorship platform with orthopedic-specific training data	Academic medical centers, tech partners	12-24 months	User satisfaction ≥4/5; mentor-mentee match rate; number of unique mentor contacts per student; perceived belonging score	NIH supplements; AAOS Foundation	High
	Deploy residency transparency dashboard with diversity metrics and match outcome data	AAOS, FREIDA, Doximity	12-24 months	Dashboard utilization rate; applicant satisfaction; data accuracy audit results; ≥70% of programs reporting	AAOS; AMA; federal transparency grants	High
	Conduct equity impact assessment and usability study before scaling	Research institutions, IRBs	12-24 months	Published equity impact assessment; usability scores by student population subgroup; identification of tool-related harms	Academic research funds	High
Phase 3: Scale	Integrate AI mentorship tools into national orthopedic pipeline programs (Perry Initiative, AAOS Nth Dimensions)	National orthopedic organizations	24-48 months	Program enrollment increase; URM match rate increase; mentor engagement rate	National organizations; philanthropy	High
	Establish longitudinal outcome tracking: match rates, career progression, workforce diversity metrics	AAOS, GME programs	24-48 months	Annual diversity report; match rate parity by race/ethnicity and institution tier; AI tool usage patterns by student population	ACGME; specialty societies	High
	Conduct randomized or prospective studies on AI mentorship tool efficacy	Academic researchers	24-60 months	Peer-reviewed publications; effect size estimates; cost-effectiveness data	NIH; AHRQ; specialty society research grants	Moderate
Phase 4: Sustain	Implement continuous algorithmic bias auditing for all AI tools	Institutional review boards, AI developers	Ongoing	Annual bias audit reports by subgroup (race, ethnicity, gender, institution tier); corrective action logs; incident reporting data	Institutional quality improvement funds	High
	Ensure human mentorship remains central; AI tools serve augmentation, not replacement	All stakeholders	Ongoing	Mentorship satisfaction surveys; documented human mentor touchpoints per student per year; qualitative assessments of mentorship quality	Embedded in existing mentorship infrastructure	High

Existing Platforms and Gaps

Several existing platforms provide partial functionality for residency program comparison. Doximity Residency Navigator and the American Medical Association's FREIDA Online database offer searchable information on program characteristics, including location, size, and some training metrics [9]. However, these platforms have significant limitations: data are often self-reported and infrequently updated, diversity metrics are inconsistently reported or absent, and match outcome data by applicant demographics are not available. A comprehensive dashboard addressing these gaps would represent a significant advance in residency selection transparency and equity.

Social media analytics for network mapping

Social media platforms have emerged as important spaces for professional networking, mentorship, and community-building in medicine. Orthopedic surgeons and trainees use Twitter/X (via hashtags such as #Orthotwitter), LinkedIn, Instagram, and TikTok to share clinical cases, discuss research findings, provide career advice, and build professional relationships that transcend institutional and geographic boundaries [24,25]. Social media analytics - computational methods for analyzing social media data to identify networks, influential users, and community structures - can help students identify potential mentors, understand professional networks, and access communities of practice that would otherwise remain invisible to those outside established networks. These tools are proposed primarily as student-facing resources for identifying potential mentors and as researcher tools for characterizing professional network structure; they are not proposed for use by residency programs for applicant screening or evaluation, which would raise serious ethical and legal concerns.

Twitter/X Network Analysis

The #Orthotwitter hashtag connects surgeons, residents, and students across institutions; network analysis enables students to identify influential mentors, map subspecialty communities, and engage strategically to build cross-institutional relationships that traditional networking cannot reach. However, Twitter/X users are not representative of the orthopedic surgery workforce. The platform over-represents surgeons who are younger, who are more comfortable with digital communication, and who practice at higher-volume academic centers. URM surgeons are systematically underrepresented on the platform, and algorithm-driven content filtering may further amplify already-prominent figures.

LinkedIn Professional Network Mapping

LinkedIn enables students to trace career pathways and identify mentors with shared backgrounds or research interests, though underrepresentation of URM surgeons and algorithm-driven content filtering may limit its utility for finding same-identity mentors. Network centrality and follower counts measure a surgeon's social media visibility, not their quality as a mentor. Any mentor identification tool must complement social media analytics with human-curated information about mentorship availability, track record, and fit, for example through structured faculty profiles maintained by orthopedic departments or specialty societies.

Instagram and TikTok Content Analysis

Instagram and TikTok content analysis offers insights into program culture, wellness, and DEI commitment that are absent from formal application materials. However, students should treat program-curated social media content as one data point among many, evaluating it alongside structured program data and, ideally, direct conversations with current residents. Critical evaluation is essential given that programs curate their social media presence.

Ethical Considerations in Social Media Analytics

Social media analytics raise important ethical considerations regarding privacy, consent, and potential surveillance. While publicly posted content is legally accessible for analysis, users may not anticipate or consent to systematic analysis of their social media activity, particularly when such analysis is used to make decisions about professional relationships or opportunities [24]. Analytics conducted on publicly posted content should use aggregate- rather than individual-level reporting, avoid linking social media presence to residency applicant evaluation, and be subject to IRB oversight when conducted for research purposes. Analytical methods must also account for platform algorithmic bias, which may marginalize URM surgeons, and intentionally prioritize diverse networks rather than reinforcing existing hierarchies. Table 2 outlines corresponding AI-based solutions for each barrier domain, including required human complements.

Benefits of AI-augmented mentorship

The following are plausible benefits based on available evidence from adjacent domains; none have been empirically demonstrated specifically in orthopedic mentorship contexts, and all require prospective evaluation before they can be considered established. Scalability, the ability to serve large numbers of users simultaneously, is a structural advantage of LLM platforms, but scalability does not equal effectiveness. A scalable tool that provides poor, biased, or generic guidance may reach more students without benefiting them [21]. Continuous asynchronous availability removes time-zone and scheduling barriers that disproportionately affect students with caregiving or work obligations [16]. Data dashboards provide standardized access to program metrics, reducing the informational advantage of elite institutions [22]. Social media analytics enable cross-institutional network building that transcends local faculty networks and geographic boundaries [20]. An important limitation of all three proposed tools is that they can expand access to information and professional visibility but cannot expand access to influence or advocacy, which are the social capital functions of sponsorship, personal introductions, and direct professional endorsement that remain gatekept by human relationships.

Limitations and challenges

AI-augmented mentorship faces limitations in three categories: technical limitations, structural access barriers, and the irreplaceable value of human mentorship.

Technical Limitations

LLMs are prone to hallucination, generating plausible-sounding but factually incorrect information, which poses risks when students rely on AI-generated guidance for high-stakes decisions such as career planning or research design. A study of LLM performance in systematic review data extraction found that while these models demonstrated high accuracy for many tasks, they also produced errors that required human oversight to detect and correct [25]. This finding underscores the critical need for user education on critically evaluating AI outputs and the importance of human oversight, particularly for consequential decisions. Algorithmic bias poses additional risks: LLMs trained on data reflecting historical inequities may perpetuate disparities in guidance or mentor matching, requiring diverse training datasets, regular bias auditing, and transparent reporting [23]. Table 4 outlines mitigation strategies.

Structural Barriers and the Digital Divide

The digital divide - disparities in access to technology, internet connectivity, and digital literacy - threatens to undermine the equity goals of AI-augmented mentorship. Students from rural areas, lower socioeconomic backgrounds, and underrepresented minority groups are more likely to lack reliable internet access, appropriate devices for accessing AI tools, and the digital literacy required to effectively utilize these technologies [16]. Without intentional efforts to address these barriers, AI-augmented mentorship risks providing additional advantages to already-privileged students while leaving behind those with limited technological access. Addressing the digital divide requires institutional provision of subsidized device and internet access, mobile-compatible AI tool design, structured AI literacy training, and national advocacy for broadband expansion in underserved areas [17]. These are not optional additions; they are preconditions for equitable implementation.

Irreplaceable Value of Human Mentorship

Perhaps the most important limitation of AI-augmented mentorship is that AI cannot replicate the relational depth, emotional support, and sponsorship that characterize effective human mentorship. Mentorship relationships provide psychosocial support, encouragement during challenging times, validation of experiences, and emotional connection that are essential for professional development and well-being [5]. AI systems, regardless of their technical sophistication, cannot provide genuine empathy, understand the nuanced emotional context of mentees' experiences, or offer the personalized encouragement that comes from a mentor who knows and believes in a mentee's potential. Sponsorship, including letters of recommendation, personal introductions, and direct advocacy, cannot be replicated by any AI system and remains the most critical function of human mentorship in a competitive field such as orthopedic surgery [5]. These limitations confirm that AI tools must complement, not replace, human mentors, who remain central to sponsorship, advocacy, and psychosocial support [5,22].

Limitations of the Review

This review has several important limitations that readers must consider when evaluating the proposed framework. First, the framework is entirely conceptual; no AI-augmented mentorship platform has been implemented, tested, or evaluated in an orthopedic surgery context, and all proposed benefits are extrapolations from adjacent fields rather than demonstrated outcomes in this population. Second, the narrative literature synthesis is not systematic; inclusion decisions reflect author judgment, and relevant literature may have been missed. Third, all cited evidence supporting AI effectiveness is drawn from other medical education domains and may not transfer to orthopedic surgery, which has a distinctive training culture, highly competitive match environment, and specific social capital dynamics. Fourth, no usability data, student perspectives, or pilot outcomes are available to validate the framework. Fifth, the proposed implementation timelines are aspirational and do not reflect formal feasibility analysis. Additional risks warrant explicit acknowledgment: students who submit AI-generated personal statements, research emails, or scholarship applications without disclosure raise authorship and authenticity concerns, and institutional AI use policies and disclosure norms must be clearly communicated before tool deployment. AI guidance is also inherently generic, a population-level approximation, whereas effective mentorship derives much of its value from contextual specificity. Students should be counseled that AI guidance is a starting point requiring human validation, not a substitute for a mentor who knows their individual circumstances.

Ethical considerations

Key ethical considerations include data privacy, algorithmic bias, informed consent, accountability, depersonalization risk, and AI transparency. Table 4 provides a comprehensive and operational overview of each domain with mitigation strategies.

**Table 4:** Ethical considerations and operational mitigation strategies for AI-augmented mentorship in orthopedic surgery AAOS, American Academy of Orthopaedic Surgeons; AI, artificial intelligence; IRB, Institutional Review Board; FERPA, Family Educational Rights and Privacy Act; HIPAA, Health Insurance Portability and Accountability Act; JAAOS, The Journal of the American Academy of Orthopaedic Surgeons; JBJS, The Journal of Bone and Joint Surgery; LLM, large language model; URM, underrepresented minority

Ethical Domain	Concern	Risk Level	Operational Mitigation Strategy	Responsible Party
Algorithmic bias	AI trained on historically biased data may perpetuate disparities in mentor matching or application evaluation	High	Annual subgroup performance audits by race, ethnicity, gender, institution tier, and socioeconomic background; bias incident reporting mechanism for students; mandatory model retraining when disparities are identified; pre-deployment equity impact assessment with URM community input	AI developers, IRBs, AAOS
Data privacy	Collection of sensitive demographic, academic, and career data raises HIPAA/FERPA concerns	High	Data minimization (collect only what is necessary); role-based access controls; retention limits (delete identifiable data after graduation or program departure); HIPAA and FERPA compliance protocols; explicit opt-in consent with plain-language explanation	Institutions, legal teams
Digital divide	Unequal access to devices, broadband, and AI platforms may worsen existing inequities	High	Subsidized device and internet access programs; offline-compatible tools; mobile-first interface design; multilingual materials; AI literacy curricula; human technical support at lower-resourced institutions; institutional provision rather than student-purchased access	Medical schools, government agencies
Informed consent	Students may not fully understand how AI tools use their data or generate recommendations	Moderate	Mandatory disclosure at point of use stating "this response was generated by an AI system"; transparent AI explainability documentation; plain-language consent forms; opt-out mechanisms; students must be told when interacting with AI vs. a human reviewer	Platform developers, institutions
Depersonalization	Over-reliance on AI may erode the human connection essential to effective mentorship	Moderate	Mandatory human mentor touchpoint requirements (minimum two documented interactions per academic year); AI tools must not substitute for required mentorship components; structured escalation protocol when students need human support	Program directors, faculty
Accountability	Unclear responsibility when AI-generated guidance leads to poor outcomes	Moderate	Clear written policies identifying responsible parties when AI advice leads to adverse outcomes; human oversight review for any high-stakes AI-generated recommendation; escalation pathway for students to request human review; documentation of AI system limitations	Institutions, AI developers, regulatory bodies
Equity paradox	AI tools designed to reduce disparities may inadvertently favor already-advantaged users	Moderate	Equity impact assessments before and after deployment; URM-centered design with community co-design requirements; comparison of outcomes by student population subgroup; iterative correction when tools disadvantage intended beneficiaries	Researchers, AI developers, URM advocates
Intellectual property	LLM-generated research support may raise authorship and plagiarism concerns	Moderate	Institutional AI use policies distributed to students before tool access; transparent disclosure requirements in publications; explicit prohibition on submitting AI-generated personal statements or essays without disclosure	Journals (JBJS, JAAOS), institutions
Surveillance	Monitoring of social media and digital behavior may feel invasive to students	Low to Moderate	Individual-level social media monitoring is prohibited without explicit, informed, and documented student consent; aggregate-only reporting for all analytics; students retain right to opt out; no analytics data shared with residency programs for applicant evaluation	Researchers, IRBs
AI transparency	Students may not know when they are interacting with AI vs. a human	Moderate	Mandatory AI disclosure at every interaction point; students retain right to request human review at any time; institutions must establish escalation pathways for human oversight of AI outputs	Platform developers, institutions
Data deletion	Students may want their data removed from AI platforms	Moderate	Clear data deletion protocols activated on student request; institutions must verify deletion within a defined timeframe; deletion rights communicated at point of consent	Institutions, legal teams

Data Privacy and Confidentiality

AI-augmented mentorship systems collect sensitive data about students' academic performance, career interests, demographic characteristics, and professional networks. Students must be informed about what data are collected, how data are used and stored, who has access to data, and how long data are retained. Students should retain the right to request deletion of their data from any AI mentorship platform, and institutions must establish data deletion protocols that can be activated on request. Consent processes must be transparent and provide meaningful opportunities for students to opt out of data collection or request data deletion. Data minimization principles, de-identification protocols, and clear institutional data governance policies are essential safeguards [18]. The collection of sensitive demographic information such as race, ethnicity, gender, sexual orientation, disability status, and socioeconomic background by AI mentorship platforms warrants specific justification. Such data should only be collected where it directly enables a demonstrably equity-relevant function, should never be shared with residency programs, and should be subject to heightened privacy protections and IRB oversight.

Algorithmic Bias and Equity

Algorithmic bias poses significant risks to equity in AI-augmented mentorship. AI systems trained on historically biased data may perpetuate disparities in mentor matching, application evaluation, or guidance provision. Addressing this risk requires proactive bias auditing, systematic evaluation of AI system outputs to identify disparities by race, ethnicity, gender, socioeconomic status, or institutional affiliation, and corrective action when biases are detected [23]. Equity impact assessments should be conducted before deploying AI-augmented mentorship systems, evaluating potential effects on different student populations and identifying risks of exacerbating existing disparities. Community co-design processes, where affected populations participate as equal partners, not merely consultants, in needs assessment, feature design, usability testing, equity impact evaluation, and ongoing governance, are a requirement, not an optional ideal.

Informed Consent and Transparency

Students should be clearly informed when they are interacting with an AI system rather than a human mentor. Mandatory AI transparency disclosures, plainly stating "This response was generated by an AI system; it should not be treated as personalized professional advice," should be required at every point of use. Informed consent requires plain-language explanation of how AI systems generate recommendations, what data are used, what biases may affect outputs, and what oversight mechanisms exist. Students should be taught to verify AI-generated information and to use these tools as one component of a broader mentorship strategy [18].

Accountability and Liability

Accountability mechanisms should include human oversight protocols that require review of AI-generated guidance for high-stakes decisions, escalation pathways that allow students to request human review when they have concerns about AI-generated outputs, and clear documentation of AI system limitations and appropriate use cases. Clear written policies must identify responsible parties when AI-generated advice leads to adverse outcomes. Institutions that adopt AI mentorship tools bear responsibility for ensuring that those tools do not generate harmful or misleading guidance. This requires pre-deployment content review, ongoing monitoring, clear reporting mechanisms for students who receive problematic outputs, and rapid-response protocols for withdrawing tools that cause demonstrable harm.

Equity implications and future directions

Equity-Centered Design

URM students, students from lower-resourced institutions, and other underrepresented groups must be involved as equal partners, not merely consultants, in the needs assessment, feature design, usability testing, equity impact evaluation, and ongoing governance of any AI-augmented mentorship platform. This is not optional: equity-centered design without equity-centered governance reproduces the structural exclusions it aims to address. Community co-design processes, where affected populations participate as equal partners in design decisions, have been shown to produce more equitable and effective systems than traditional top-down design approaches. Design must also prioritize accessibility for users with limited internet access or digital literacy, with diverse user testing to identify barriers that disproportionately affect underrepresented students [18,19].

Implementation Framework and Outcome Tracking

Table 3 outlines a recommended implementation framework for AI-augmented mentorship programs in orthopedic surgery, specifying phases, recommendations, responsible stakeholders, timelines, measurable outcomes, funding sources, and priorities. This framework emphasizes the importance of establishing foundational infrastructure, standardized diversity data reporting, equity-centered design guidelines, and digital literacy curricula before deploying AI tools at scale. Phase 2 piloting must include a formal equity impact assessment and usability evaluation before national scaling is recommended in Phases 3 and 4. Longitudinal outcome tracking should include match rates by race, ethnicity, gender, and school tier; AI tool usage patterns by student population; and workforce diversity metrics over time [22].

Future Research Directions

Near-term research priorities should include prospective usability studies evaluating accessibility, trust, perceived belonging, and satisfaction among diverse student populations; pilot studies measuring mentor access, contact quality, and research engagement as early process outcomes; and equity impact assessments comparing outcomes by race/ethnicity, gender, institution tier, and socioeconomic background before broader deployment. Randomized controlled trials comparing AI-augmented with traditional mentorship are a longer-term priority and should follow, not precede, foundational pilot data establishing that the tools work and do not cause harm [5,22].

National organizational leadership

Realizing the potential of AI-augmented mentorship to increase diversity in orthopedic surgery requires leadership from national organizations. Proposed role delineations include AAOS, which should establish standardized diversity data reporting requirements and equity-centered AI design guidelines; AOA, which should facilitate cross-institutional mentor network development; ACGME, which should mandate minimum AI literacy training in residency program requirements and monitor compliance with diversity data reporting; ABOS (American Board of Orthopedic Surgery), which should incorporate DEI metrics into program accreditation assessment; medical schools, which should provide AI tool access, AI literacy training, and technical support as part of standard student support infrastructure; residency programs, which should designate human mentors to work alongside AI tools and establish escalation protocols; and student organizations including SNMA (Student National Medical Association) and resident councils, which should participate in tool co-design, evaluation, and governance. We recommend that national organizations prioritize establishing evidence-based standards and equity guidelines for AI mentorship tools rather than hosting proprietary platforms directly. A standard-setting role scales more readily across institutions, and it avoids the conflict of interest that arises when one organization both sets the standards for AI mentorship tools and operates the platforms being measured against them. These recommendations assume organizational will, data-sharing agreements across institutions, sustained funding, and resolution of FERPA (Family Educational Rights and Privacy Act) and HIPAA (Health Insurance Portability and Accountability Act) compliance questions, none of which are trivial.

Professional societies must also drive cultural change by educating faculty on AI tool integration and establishing expectations that programs provide both human mentorship and AI resources [5].

## Conclusions

Orthopedic surgery faces a persistent diversity crisis that traditional mentorship models have been insufficient to resolve. Women and racial and ethnic minorities remain persistently underrepresented in orthopedic surgery, and match disparities continue to fall hardest on URM students and those at lower-resourced institutions. The conceptual framework presented in this paper proposes three complementary AI-augmented approaches that may help address specific informational and geographic barriers. LLM-based virtual mentorship provides scalable, 24/7 access to career guidance, research support, and application strategy that can supplement, though not replace, access to human mentors. Residency transparency dashboards may level the informational playing field by giving all applicants standardized access to program diversity metrics, training quality indicators, and match outcomes. Social media analytics may help students identify potential mentors and communities of practice across demographic and institutional boundaries. Together, these tools address the geographic isolation, information asymmetry, and social capital deficits that have historically excluded talented students from the specialty. However, their impact is unproven in this context, and implementation without rigorous evaluation risks doing harm. Successful implementation requires confronting three critical limitations with equal rigor. The digital divide threatens to reproduce existing inequities if URM students and those from lower socioeconomic backgrounds lack the devices, connectivity, and digital literacy to access AI tools, making subsidized access programs and accessible interface design non-negotiable preconditions. Algorithmic bias risks encoding historical disparities, requiring diverse training datasets, regular bias auditing, and transparent reporting as ongoing institutional commitments rather than one-time checks. Most fundamentally, AI cannot replicate the relational depth, psychosocial support, or sponsorship that effective human mentors provide, and these tools must augment rather than replace human relationships.

National leadership from the AAOS, AOA, and ACGME is essential to operationalize the phased implementation framework proposed here, establishing standardized diversity data reporting requirements, equity-centered AI design guidelines, and longitudinal workforce tracking that hold programs accountable over time. The goal is a comprehensive mentorship ecosystem where every student, regardless of institutional affiliation, geographic location, or social capital, has access to the guidance and networks needed to pursue a career in orthopedic surgery. A more diverse orthopedic workforce will better serve increasingly diverse patient populations, bring varied perspectives to research and innovation, and strengthen the profession's capacity to address complex musculoskeletal health challenges. AI-augmented mentorship, when thoughtfully designed, rigorously evaluated, and equitably implemented, may help reduce selected informational, geographic, and network-access barriers, but only if implemented with equity-centered design, human mentorship, bias monitoring, and strong data governance. The goal is not to substitute AI for human mentorship but to ensure that no student is excluded from the guidance needed to pursue orthopedic surgery simply because of where they trained.
